# Angle Kappa agreement between Scheimpflug tomography, combined placido Scheimpflug and combined slit scanning placido systems

**DOI:** 10.1007/s10792-022-02433-z

**Published:** 2022-07-29

**Authors:** Mo’mena Ahmad A. Awad-Allah, Hesham Mohamed Gharieb, Rania Gamal Eldin Zaki, Ihab Saad Othman

**Affiliations:** 1 Ain Shams University, Cairo, 11727 Egypt; 2 Cairo University, Cairo, Egypt

**Keywords:** Kappa angle, Pentacam, Orbscan III, Sirius device

## Abstract

**Purpose:**

To compare the measured or calculated angle Kappa using Oculus pentacam HR, Sirius and Orbscan III devices.

**Patients and methods:**

A prospective randomized cohort study, conducted on 47 eyes of 47 healthy orthotropic individuals, with an age range of 18–50 years and a corrected Snellen’s distance visual acuity (CDVA) of 0.8 decimal or better. Angle Kappa is assessed directly using Orbscan® III software version 1.8.165.1. (Bausch and Lomb Rochester, New York, United States), while Pentacam® HR 1.21r.65 (Oculus Optikgeräte GmbH, Wetzlar, Germany) and Sirius device (CSO, version 3.2.1.60, Costruzione Strumenti Oftalmici, Florence, Italy) were used to calculate angle kappa indirectly.

**Results:**

Least mean difference of estimated angle Kappa was between Orbscan and Pentacam devices (− 0.18° ± 1.8), and it was statistically insignificant (*p* value = 0.1294). Differences between both Orbscan and Sirius, and Pentacam and Sirius were statistically significant (*p* value = 0.0004 and < 0.0001 consecutively). Bland Altman analysis showed a 95% confidence interval between Orbscan III and Pentacam of − 3.76 to 3.4 and between Orbscan III and Sirius of − 3.79 to 2.26.

**Conclusion:**

Pentacam parameters can be used as a reliable method to calculate angle kappa indirectly, without usage of any additional measurements from other machine. Sirius device parameters could also be used, but with less accurate results. A simple modification to those devices’ software to calculate it, and incorporate it in the printout is possible, and highly recommended.

## Introduction

Angle Kappa is defined as the angle between the visual axis and pupillary axis. The visual axis is a theoretical axis line connecting the fixation point with the foveola, passing through the two nodal points of the eye. Pupillary axis is a line perpendicular to the cornea, which passes through the center of the pupil, and is identified by the first Purkinje image [1]. Angle Kappa is either positive (nasal light reflex), or negative (temporal light reflex). An average positive angle of 5° is found in most emmetropes [2].

Classically, in clinical practice angle Kappa is considered in squint surgeries decisions in strabismic patients with large angles [3]. However, with the exponential growth of the number of patients undergoing refractive surgery, the importance of an accurate estimation of this angle increases. Angle Kappa is important for the ablation centration, to avoid complications such as the decreased safety, and the increased likelihood of irregular astigmatism [4]. For example, the hyperopic eyes are known to have a larger angle Kappa in comparison to myopes, thus, even a little decentration can significantly affect the outcome due to the increased high-order aberration and coma aberration. This may lead to several postoperative complications including visual loss, glare, and poor night vision [5]. The angle Kappa measurement is also vital for the ideal positioning of phakic and multifocal intraocular lenses (IOLs).

The aim of our study is to compare the measured or calculated angle Kappa using Orbscan III, Pentacam, and Sirius devices.

## Subjects and methods

### Design and subjects

This study is a prospective randomized cohort, conducted on 47 eyes of 47 healthy orthotropic individuals. The study was approved by Cairo University Ethical Committee and was adherent to the tenets of the Declaration of Helsinki. The intended examination and investigations were explained in simple words for all the subjects, then, a written informed consent was obtained. The study was conducted at “Eye World Hospital” in Giza, Egypt. Inclusion criteria were subjects’ age range between 18 and 50 years and a corrected Snellen’s distance visual acuity (CDVA) of 0.8 decimal or better. Exclusion criteria were history of any deviation or strabismus, with or without orthoptic or surgical treatment; any intraocular, corneal, or refractive surgery; contact lens usage for the past 3 weeks; any corneal anomaly; any ophthalmic or systemic drug consumption, and severe dry eye.

### Methods

The assessment included unaided visual acuity (UAVA) and best corrected visual acuity (BCVA) using Snellen’s charts. Manifest refraction was measured with the Potec PRK-7000 Autorefractor/Keratometer (Potec Co. Ltd, Daejeon, Korea). A clinical examination with the slit-lamp biomicroscopy, including assessment of the posterior segment with 90D lens (Volk Optical, USA), was done to all patients with dilated fundus examination.

The patients’ angle Kappa was assessed directly using the Orbscan® III software version 1.8.165.1. (Bausch and Lomb Rochester, New York, United States). The Pentacam® HR 1.21r.65 (Oculus Optikgeräte GmbH, Wetzlar, Germany) and the Sirius devices (CSO, version 3.2.1.60, Costruzione Strumenti Oftalmici, Florence, Italy) were used to calculate angle kappa indirectly using the geometrical model proposed by Sung et al. This model depends on the fact that the Pentacam machine parameters can be used to identify the distance between the center of the pupil and the corneal reflex point, and this distance, together with the anterior chamber depth (ACD) are converted to the angle lambda using the second law of cosines. And since angle lambda and angle kappa are almost equal, the calculated angle was considered equivalent to angle kappa [6]. A similar model is used for the Sirius device.

The three investigations were done at the same session under the same lighting conditions by a single experienced operator. They were all done before the previously mentioned examination, starting with the Orbscan III device. In an order according to the patient cycle: Orbscan 3 1st then Sirius then Pentacam, all within one hour. The subjects were well instructed on the straight head positioning and gaze. Three images were taken for each eye, and the one with the best quality was used.

### Statistics analysis

The data were retrieved from the Pentacam and the Sirius devices as comma-separated values (.csv) files, and converted into Excel spreadsheet (.xlsx) files by Microsoft Excel 356 program (Redmond, Washington, USA).

The data were analyzed by MedCalc Statistical Software version 18.9.1 (MedCalc Software bvba, Ostend, Belgium). The data were described as mean, range and 95% confidence interval (CI). Test of Normality was done using Kolmogorov Smirnov test. The comparisons between the three modalities of angle Kappa assessment were done by independent t-test, where *p* ≤ 0.05 was set to be of the significant level. Anova test with Posthoc analysis was done for difference between groups. A Bland Altman plot was also performed.

## Results

The study was conducted on 47 eyes of 47 patients, 25 males (53.2%) and 22 females (46.8%). There were 24 right eyes (51.1%) and 23 left eyes (48.9%). The demographic data of the patients are shown in Table 1.

**Table 1:** The demographic data

	Mean	Range	95% CI
Age	30.36 ± 7.37	(19–49)	28.198–32.526
Sphere	− 3.57 ± 1.95	(0 to − 7.5)	− 4.160 to − 2.975
Cylinder	− 1.09 ± 1.03	(1.5 to − 4.00)	− 1.387 to − 0.783
SE	− 4.13 ± 2.02	(0 to − 8.50)	− 4.737 to − 3.512
UCVA	0.11 ± 0.16	(0.01–0.8)	0.0416–0.173
BCVA	0.98 ± 0.19	(0.4–1.5)	0.921–1.037
AL	23.8 ± 0.77	(22.13–26.3)	23.62–24.06

*SE* spherical equivalent, *UCVA* uncorrected visual acuity, *BCVA* best corrected visual acuity, *AL* axial length

**Table 2:** The data obtained by Pentacam, Orbscan III, and Sirius devices

	Pentacam	Orbscan III	Sirius
K1	42.9 ± 1.49 (40.7–45.7)	42.9 ± 1.51 (40.70–45.9)	42.9 ± 1.51 (40.62–45.6)
K2	44.2 ± 1.73 (41.5–47.4)	44.3 ± 1.65 (41.80–47.3)	44.2 ± 1.68 (41.27–47.0)
*Q* value front	− 0.3 ± 0.14 (− 0.57 to 0.2)	− 0.3 ± 0.18	− 0.12 ± 0.13 (0.17 to − 0.38)
Pupil center *X*-coordinate	− 0.01 ± 0.15 (− 0.51 to 0.4)		− 0.03 ± 0.16 (0.28 to − 0.39)
Pupil center *Y*-coordinate	0.01 ± 0.12 (− 0.3 to 0.3)		− 0.02 ± 0.14 (0.24 to − 0.47)
CCT	533 ± 25 (483–588)	540 ± 27 (481–610)	525 ± 76 (56–598)
Thinnest Location	527 ± 25 (474–586)	531 ± 28 (471–601)	528 ± 28 (464–595)
ACD	3.6 ± 0.39 (2.66–4.3)	3.4 ± 1.30 (2.03–4.01)	3.2 ± 0.25 (2.51–3.8)
Corneal diameter	11.9 ± 0.39 (11.3–12.9)	11.5 ± 1.35 (2.90–12.6)	12.2 ± 0.35 (11.61–12.9)
Pupil diameter	3.1 ± 0.63 (2.02–4.7)	3.2 ± 0.81 (− 0.30 to 4.6)	3.4 ± 0.55 (2.47–5.2)
Angle Kappa	2.4 ± 1.69 (0.16–9.9)	2.2 ± 1.17 (0.33–5.1)	2.9 ± 1.74 (0.86–8.6)
Kappa intercept *X*		− 0.02 ± 0.32 (− 0.80 to 0.6)	− 0.01 ± 0.37 (0.69 to − 0.76)
Kappa Intercept *Y*		0.12 ± 0.49 (− 0.50 to 3.1)	− 0.006 ± 0.35 (0.63 to − 0.99)

K1, flat keratometry reading; K2, steep keratometry reading; CCT, central corneal thickness; ACD, anterior chamber depth

A summary of the data obtained from the three devices is shown in Table 2 The table also contains the measured angle kappa by the Orbscan (2.2 ± 1.17), and the calculated angle kappa by the Pentacam (2.4 ± 1.69) and the Sirius devices (2.9 ± 1.74).

A paired t-test was done to compare the devices’ measured or estimated angle kappa. The least mean difference was between the Orbscan III and the Pentacam devices (− 0.18 ± 1.8), and it was statistically insignificant with a *p* value of 0.1294. The differences between both the Orbscan and the Sirius, and the Pentacam and the Sirius were statistically significant, with a *p* value of 0.0004 and < 0.0001consecutively (Table 3).

**Table 3:** Paired t-test for the measured or estimated angle kappa by the three devices

	Paired *t*-test	Mean difference ± SD (95% CI)	*r*	*p* value
Orbscan Ka–Pentacam Ka	0.509	− 0.18 ± 1.8 (− 0.71 to 0.36)	0.224	0.1294
Orbscan Ka–Sirius Ka	0.001	− 0.77 ± 1.5 (− 1.22 to − 0.31)	0.499	0.0004*
Pentacam Ka–Sirius Ka	0.002	0.59 ± 1.2 (0.22 to 0.95)	0.737	< 0.0001*

*Ka* angle kappa

^*^Refers to statistically significant *p* values (*p* ≤ 0.05)

ACD was tested between 3 machines (as it was used for calculating Angle kappa by Pentacam and Sirius machines). ANOVA test showed no statistical significance within groups (*p* = 0.091).

**Fig. 1:**
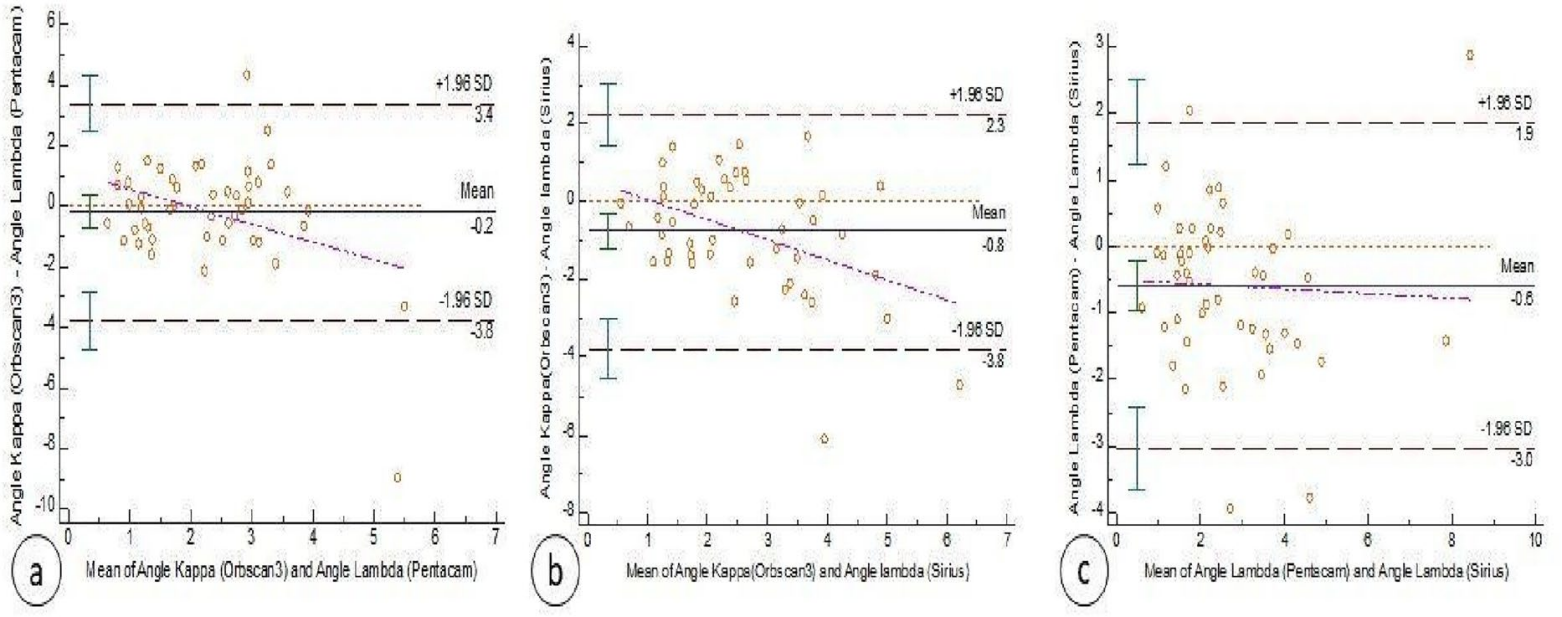
Bland Altman analysis plot comparing the three machines. **a** Plot comparing Orbscan III measured angle kappa and Pentacam calculated angle Kappa, **b** plot comparing Orbscan III measured angle kappa and Sirius calculated angle Kappa, **c** plot comparing calculated angle kappa by both Pentacam and Sirius devices

Bland–Altman plots of the paired angle kappa differences against the mean values and the 95% LoA are shown in Fig. 1a–d. The 95% LoA was 3.4° to -3.8° between Orbscan III and Pentacam (Fig. 1a), 2.3° to -3.8° between Orbscan III and Sirius (Fig. 1b) and 1.8° to -3° between Pentacam and Sirius (Fig. 1c).

## Discussion

Our study aimed at calculating angle kappa, using two widely used machines in the settings of refractive surgery, the Pentacam and the Sirius devices. Calculated angle kappa was then verified by comparing it to the measured angle kappa by Orbscan III device.

According to Le Grand and El Hage [7], angle kappa is defined as the angular distance between the line of sight (the line that connects the pupillary center and the fixation point) and the pupillary axis. This definition makes the angle measurable in contrary to the original definition by Landolt [8].

The conventional method for the assessment of this angle used to be the synaptophore, but with the advance of different automated machines, angle kappa could be measured by Orbscan, OPD scan, and Galilei. The Orbscan II device and the later models identify the center of the pupil, and finds where the axis perpendicular to this center intercepts the cornea. It can automatically determine angle kappa with a special software that measures the distance between the center of the pupil and the center of the placido ring (which represents the axis of sight), which was reflected on the cornea [9].

One of the most important clinical applications of angle kappa is the centration in refractive surgery, either the centration of ablation in laser based procedures, or the centration of phakic and multifocal IOLs. Since the aforementioned devices that can measure angle kappa directly are not always available, an alternate method using the more commonly used devices as the Pentacam and the Sirius devices is highly needed. Sung et al. described in 2015 a method to indirectly identify angle kappa. The method depends on identifying two parameters, the anterior chamber depth (ACD), and the distance between the points for the pupil center intercept with the cornea and the corneal vertex. This distance could be calculated from the pupil center x- and y-coordinates. Those two values, using the second law of cosines, are used to calculate angle kappa [6]. In our study the angle kappa measured by Orbscan III was 2.2 ± 1.17. Angle kappa is postulated to have a relation with the race. We couldn’t find in the literature a study among the Egyptian population to measure it, but we found studies conducted on Iranians, a middle eastern population. Gharaee et al. [10] found a mean measurement of angle kappa of 4.97° ± 1.38° measured by Orbscan II. Basmak et al. [9] observed a similar average angle kappa of 5.22°. Both values are almost the double of the value we found. This could be attributed to the racial difference or the fact that the average spherical error among our sample is of -3.57 ± 1.95. It is known that myopes tend to have a smaller angle kappa [11].

In our study, the mean difference (assessed by *t*-test) between the angle kappa measured by Orbscan III and the calculated from the Pentacam parameters was statistically insignificant (*p* value = 0.1294). Yeo et al. had a highly statistically significant difference between Orbscan II and Pentacam, with a *p* value < 0.001. This could be explained by their usage of ultrasound biomicroscopy (UBM) to assess the ACD (not the ACD measured by Pentacam). There was a highly statistically significant difference between the ACD measured by UBM and Orbscan II. In our study, there is a slight difference between the measurement of ACD obtained by Pentacam and Orbscan III.

The importance of this study is that it postulates that the Pentacam parameters could be used to calculate angle kappa with accuracy near that of the Orbscan III device. The calculation was done using a simple equation that could be incorporated easily in the software of the machine by the manufacturers. The Sirius device parameters-based calculation of angle kappa was less accurate, yet, it could be used to get a rough estimation. Further studies using a larger sample size, and a stratification of the sample according to age groups and refraction could be conducted to verify the results of this study, and to encourage the manufacturers to incorporate the afore mentioned modification into the software which would be an added value to the usage of these machines.

## Conclusion

The Pentacam parameters could be a reliable method of calculating angle kappa indirectly without the use of any additional measurements from any other machines. The Sirius device parameters could also be beneficial, but with less accurate results.

## Limitation of the study

Hyperopic patients were excluded from the study as there was in enough data concerning them, so we had to exclude this data to avoid false statistics. We recommend conducting a separate study concerned with hyperopic patients.
